# Isomalto oligosaccharide sulfate inhibits tumor growth and metastasis of hepatocellular carcinoma in nude mice

**DOI:** 10.1186/1471-2407-11-150

**Published:** 2011-04-22

**Authors:** Chun-Li Xiao, Zhong-Hua Tao, Lin Guo, Wei-Wei Li, Jin-Liang Wan, Hui-Chuan Sun, Lu Wang, Zhao-You Tang, Jia Fan, Wei-Zhong Wu

**Affiliations:** 1 Liver Cancer Institute and Zhongshan Hospital, Fudan University, Key Laboratory of Carcinogenesis and Cancer Invasion, Ministry of Education, Shanghai 200032, China; 2 Department of Clinical Laboratory, Cancer Hospital, Fudan University, Shanghai 200032, China; 3 Institute of Biomedical Sciences of Fudan University, Shanghai 200032, China

**Keywords:** Isomalto oligosaccharide sulfate, hepatocellular carcinoma, proliferation, metastasis, apoptosis

## Abstract

**Background:**

Hepatocellular carcinoma (HCC) usually has a dismal prognosis because of its limited response to current pharmacotherapy and high metastatic rate. Sulfated oligosaccharide has been confirmed as having potent antitumor activities against solid tumors. Here, we explored the preclinical effects and molecular mechanisms of isomalto oligosaccharide sulfate (IMOS), another novel sulfated oligosaccharide, in HCC cell lines and a xenograft model.

**Methods:**

The effects of IMOS on HCC proliferation, apoptosis, adhesion, migration, and invasiveness in vitro were assessed by cell counting, flow cytometry, adhesion, wound healing, and transwell assays, respectively. The roles of IMOS on HCC growth and metastasis in xenograft models were evaluated by tumor volumes and fluorescent signals. Total and phosphorylated protein levels of AKT, ERK, and JNK as well as total levels of c-MET were detected by Western blotting. IMOS-regulated genes were screened by quantitative reverse-transcription PCR (qRT-PCR) array in HCCLM3-red fluorescent protein (RFP) xenograft tissues and then confirmed by qRT-PCR in HepG2 and Hep3B cells.

**Results:**

IMOS markedly inhibited cell proliferation and induced cell apoptosis of HCCLM3, HepG2, and Bel-7402 cells and also significantly suppressed cell adhesion, migration, and invasion of HCCLM3 in vitro. At doses of 60 and 90 mg/kg/d, IMOS displayed robust inhibitory effects on HCC growth and metastasis without obvious side effects in vivo. The levels of pERK, tERK, and pJNK as well as c-MET were significantly down-regulated after treatment with 16 mg/mL IMOS. No obvious changes were found in the levels of pAkt, tAkt, and tJNK. Ten differentially expressed genes were screened from HCCLM3-RFP xenograft tissues after treatment with IMOS at a dose of 90 mg/kg/d. Similar gene expression profiles were confirmed in HepG2 and Hep3B cells after treatment with 16 mg/mL IMOS.

**Conclusions:**

IMOS is a potential anti-HCC candidate through inhibition of ERK and JNK signaling independent of *p53 *and worth studying further in patients with HCC, especially at advanced stages.

## Background

Hepatocellular carcinoma (HCC) is the sixth most common cancer and the third leading cause of cancer-related death globally [1]. As indicated in statistics, the disease is diagnosed in 30% to 40% of all patients at early stages and about 20% of all patients are amenable to curative therapies, such as resection, liver transplantation, and radiofrequency ablation [2,3]. Five-year survival rates of up to 60% to 70% have been achieved in well-selected patients [2]. However, HCC at advanced stages usually carries a dismal prognosis because of liver dysfunction, lack of effective treatment options, and a high metastatic rate [4,5]. Therefore, it is urgent to explore new therapeutic options for patients with advanced HCC.

Heparanase inhibitor has recently become an attractive agent for highly malignant tumors, due to its antiangiogenic and antimetastatic activities [6-10]. Two representatives, phosphomannopentaose sulfate (PI-88) and oligomannurarate sulfate (JG3), were reported to have inhibitory effects on tumor growth and metastasis [11,12]. Phase 1 and 2 trials of PI-88 have been finished and have shown potential antitumor effects [13-16].

Two distinctive differences in molecular structure exist between isomalto oligosaccharide sulfate (IMOS) and PI-88. IMOS is composed of four sulfated isomaltose molecules with a molecular weight <1500 Da, whereas PI-88 is composed of five sulfated mannose molecules with a molecular weight of 2100 to 2585 Da. Such alterations in structure may affect its toxicity and antitumor effects. In this report, we present our preliminary evidence of the effects of IMOS on experimental HCC growth and metastasis.

## Methods

### IMOS

**Figure 1:**
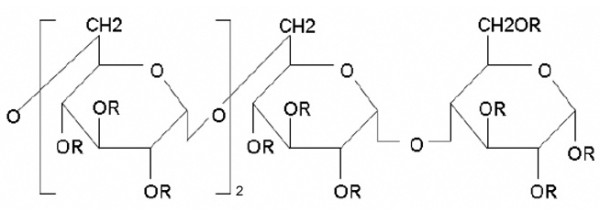
**Chemical structure of IMOS**. R: SO_3_Na or H

IMOS, with a patent (patent no. ZL2005 1 0002141.8) granted by the State Food and Drug Administration of China, is designed and successfully synthesized de novo by Herbon Polysaccharide Bio-tech. Figure 1 shows the chemical structure of IMOS. IMOS was dissolved in Dulbecco modified Eagle medium (DMEM) containing 10% fetal bovine serum (FBS; Gibco BRL, Grand Island, NY, USA), sterilized with a 0.22-μm filter (Millipore, Billeria MA, USA), and reserved at a concentration of 320 mg/mL for in vitro assays. In a similar way, IMOS was dissolved in saline under sterile conditions with a concentration of 600 mg/mL for in vivo assays.

### Cell lines

Four human hepatoma cell lines HepG2, Bel-7402, Hep3B, HCCLM3, and its red fluorescent protein (RFP)-expressing derivative, HCCLM3-RFP, were used in this study [17]. HCCLM3, HCCLM3-RFP, and HepG2 were cultured in DMEM, Hep3B in minimum essential medium (MEM), and Bel-7402 cells in RPMI-1640, supplemented with 10% FBS containing 5% CO_2 _at 37°C.

### Cell proliferation assay

Cell proliferation was assessed by the method described previously [17]. In brief, HCCLM3, HepG2, and Bel-7402 cells were seeded into 96-well plates at 2 × 10^3 ^cells/well. Twenty-four hours later, cells were exposed to IMOS at doses ranging from 0 to 64 mg/mL. On days 1, 2, 3, 4, and 5, cells were digested with pancreatic enzymes including ethylenediaminetetraacetic acid (EDTA) and washed with phosphate-buffered saline (PBS). Cell numbers were then counted by the Countess™ automated cell counter (Life Technologies, CA).

### Cell cycle and apoptosis assays

Cell cycle and apoptosis were detected using the Annexin V-FITC Apoptosis Detection Kit™ according to the manufacturer's instructions (BD Pharmingen, San Diego, CA). Briefly, HCCLM3, HepG2, and Bel-7402 cells were plated into 6-well plates at 4 × 10^5 ^cells/well. After treatment with IMOS at 0, 2, 4, 16, 32, or 64 mg/mL for 24 hours, the cells were fixed with ethanol and stained with annexin V for early apoptosis assay by a fluorescence-activated cell sorter (FACS) Calibur cytometer (BD Biosciences, San Jose, CA, USA). In the same way, 72 hours after IMOS treatment, the cells were stained with propidium Iodide (PI) for late apoptosis and cell cycle assays.

### Cell adhesion assay

The 96-well flat-bottom plates were precoated with 50 μL/well of 1:8 PBS-diluted Matrigel at 4°C overnight. After removing all coating solutions, the plates were blocked with 150 μL of 1% bovine serum albumin for 1 hour at 37°C. Then, HCCLM3 cells were treated with 0, 16, 32, or 64 mg/mL IMOS for 4 hours, seeded into Matrigel-coated wells at 5 × 10^4 ^cells/well, and incubated for 2 hours at 37°C in 5% CO_2_. After extensive washing, cells were fixed with 100 μL/well of 4% formaldehyde for 20 minutes and stained with a hematoxylin solution for 10 minutes. The average numbers of adhesion cells in four quadrants were counted by inverted microscope.

### Wound healing assay

Cell migration was analyzed by a wound healing assay. When cells grew to 90% of confluency, a scratch wound in the monolayer was made using a pipette tip. After washing away all detached cells with PBS, the remaining cells were treated with 0, 16, 32, or 64 mg/mL IMOS and then the distances of wounds were measured by microscope at 0, 24, and 48 hours after treatment. Cell motility was evaluated the following formula: Cell motility = (distance_24 or 48 hours _- distance_0 hour_)/distance_0 hour_.

### Invasion assay

Cell invasion was analyzed by a Transwell™ Permeable Supports system (Corning, Inc., Corning, NY, USA) according to the manufacturer's instructions. HCCLM3 cells were pretreated with 0, 16, 32, or 64 mg/mL IMOS for 48 hours, and then seeded into the Matrigel-coated upper insert at 8 × 10^4 ^cells/24-wells in medium supplemented with 1% serum. Medium containing 10% serum was added to the well as a chemoattractant. Following a culture of 48 hours, non-invading cells were removed from the upper surface by wiping with a cotton swab. The membrane was fixed with 4% formaldehyde for 15 minutes at room temperature. The invading cells were stained with Giemsa (Sigma, Munich, Germany) for 25 minutes, and their numbers in 10 fields of each triplicate filter were analyzed by inverted microscope.

### Protein levels detected by Western blotting

Total and phosphorylated protein levels of AKT, ERK, and JNK as well as total protein of c-MET in HepG2 and Hep3B cells were evaluated by Western blotting. About 20 μg protein was extracted from sham-treated and 16 mg/mL IMOS-treated cells, separated by 10% sodium dodecyl sulfate-polyacrylamide gel electrophoresis (SDS-PAGE), transferred onto polyvinylidene fluoride membranes, and then reacted with primary rabbit antibodies against total and phosphorylated AKT, ERK, and JNK(1:500; Bioworld Tech, Minneapolis, MN, USA), c-MET (1:1000, Epitomics, Burlingame, CA, USA) and glyceraldehyde-3-phosphate dehydrogenase (GAPDH). After being extensively washed with PBS containing 0.1% Triton X-100, the membranes were incubated with alkaline phosphatase-conjugated goat anti-rabbit antibody for 30 minutes at room temperature. The bands were visualized using 1-step™ NBT/BCIP reagents (Thermo Fisher Scientific, Rockford, IL, USA) and detected by the Alpha Imager (Alpha Innotech, San Leandro, CA, USA).

### Tolerable dose assay of IMOS in vivo

A dose-escalation strategy was used in male athymic BALB/c mice (Institute of Materia, CAS, Shanghai, China) to determine maximum tolerable dose of IMSO. Eighty mice, aged 4 weeks, were divided into groups of 10 mice apiece, with each mouse intraperitoneally injected with IMOS at a dose of 0, 30, 60, 90, 180, 360, 480, or 600 mg/kg/d, respectively. Mouse survival was monitored every day. We planned to halt the dose escalation would be halted if mice died. Serum was collected for assays of hepatorenal function. Plasma was collected for determining thrombocyte counts. Heart, liver, and kidney tissues were subjected to hematoxylin and eosin staining for pathologic examinations. The maximum nonlethal dose was determined for the following in vivo therapeutic study. All procedures were approved by the Animal Care and Use Committee of Shanghai, China.

### Antitumor growth and metastasis assays in vivo

Antitumor activities of IMOS in vivo were assessed against HCCLM3-RFP xenografts. Three 4-week-old male athymic BALB/c mice were injected subcutaneously with 1 × 10^7^/0.2 mL of HCCLM3-RFP cells in the right upper flank region to establish subcutaneous xenograft models. Four weeks later, the tumors that had grown to 1 cm in diameter were removed, cut into 1-mm^3 ^pieces, and implanted into livers of another 24 mice to establish orthotopic xenograft models as described previously [18]. Then, all mice were randomly divided into four groups of six mice each and intraperitoneally injected with IMOS at 0, 30, 60, or 90 mg/kg/d once daily for 30 consecutive days. Fluorescent images of in situ tumor were taken once a week with the mouse anesthetized with 50 mg/kg sodium pentobarbital. On day 30, all mice were sacrificed and tumor volume was calculated using the formula V (mm^3^) = width^2 ^(mm^2^) × length (mm)/2. Metastatic foci in lungs and mesenteries were counted by fluorescent stereomicroscope (stereomicroscope: Leica MZ6; illumination: Leica L5 FL; C-mount: 0.63/1.25; CCD: DFC 300FX). Fluorescence area (AOI, pixel) was quantified by Image-Pro Plus 6.0 (Media Cybernetics, Silver Spring, MD, USA) as described previously [17].

### IMOS-regulated genes detected by quantitative reverse-transcription polymerase chain reaction (qRT-PCR) and qRT-PCR array

Tumor tissues from mice treated with IMOS at a dose of 0 or 90 mg/kg/d were enrolled for differentially expressed gene analysis by RT Profiler PCR Arrays (SABioscience, PAHS-027A, Frederick, MD, USA) and performed by Kangchen Bio-tech (Shanghai, China). The mRNA levels of differentially expressed genes in HepG2 and Hep3B cells with 16-mg/mL IMOS treatment were confirmed by qRT-PCR. Total RNA of cells was extracted using RNeasy MinElute Cleanup Kit (Qiagen, Valencia, CA, USA). Then, 1.5 μg RNA was reversely transcribed into first-strand cDNA using SuperScript™ III Reverse Transcriptase (Invitrogen, NY, USA). Primer sequences and amplification conditions are listed in Additional file 1. The reactions were performed on a DNA Engine Opticon system (MJ Research, Reno, NV, USA) using SYBR^® ^Green PCR Master Mix (Applied Biosystems). Following each cycle, SYBR green fluorescence was monitored and the melting curve was analyzed to ensure that a single PCR product was obtained. Afterward, the size and specificity of amplicons were confirmed by 2.5% agarose gel electrophoresis. All reactions were repeated in three separate runs and evaluated with the Opticon Monitor software (Version 1.02). GAPDH was used to normalize the samples. RNase-free water (Qiagen) was included as a negative control in RNA extraction and in each run.

### Statistical analysis

Statistical analysis was performed with SPSS 15.0 for windows (SPSS, Chicago, IL, USA). Quantitative variables were expressed as means ± SD and analyzed by ANOVA. Results were considered statistically significant at *P *< 0.05.

## Results

### Inhibitory effects of IMOS on HCC proliferation

**Figure 2:**
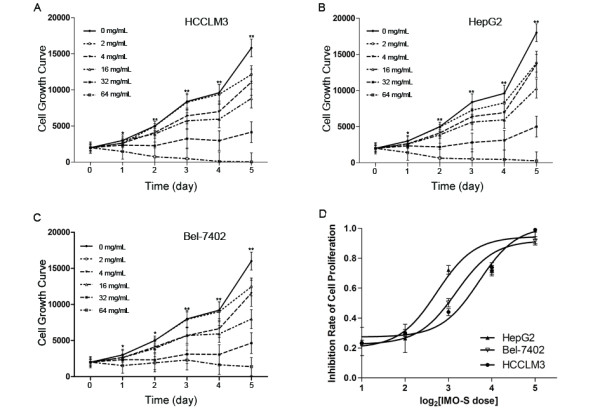
**Effects of IMOS on proliferation of HCC cells**. Proliferation of HCCLM3, HepG2, and Bel-7402 cells was significantly inhibited by IMOS in a dose-dependent manner, especially when exposed to 16- and 64-mg/mL doses of IMOS. (A-C) Growth curve of HCCLM3, HepG2, and Bel-7402 cells, respectively, treated with IMOS at doses ranging from 0 to 64 mg/mL for 5 consecutive days. (D) Inhibition ratio of proliferation of HCCLM3, HepG2, and Bel-7402 cells was markedly increased after ≥4 mg/mL IMOS treatment.

To explore the effects of IMOS on HCC proliferation, HCCLM3, HepG2, and Bel-7402 cells were treated with IMOS at doses ranging from 0 to 64 mg/mL. IMOS dramatically decreased cell numbers of all tested cell lines in a dose-dependent manner, especially when exposed to 16- and 64-mg/mL doses of IMOS (Figure 2A-C). The inhibitory ratio of IMOS on cell proliferation was significantly increased from 23.3% ± 1.9% to 99.06% ± 4.6% in HCCLM3 cells, from 24.4% ± 9.5% to 98.5% ± 9.8% in HepG2 cells, and from 27.8% ± 1.2% to 91.4% ± 1.6% in Bel-7402 cells during a 5-day treatment (Figure 2D). The data suggest IMOS has robust suppressive effects on HCC proliferation.

### Cell cycle arrest and apoptosis induced by IMOS

**Figure 3:**
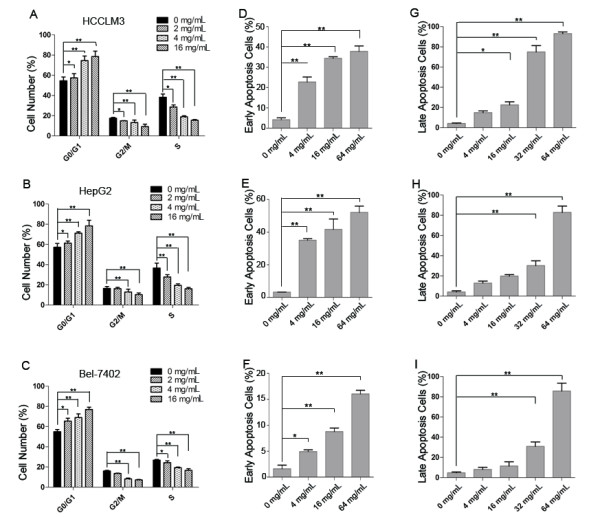
**Cell cycle arrest and apoptosis induced by IMOS in HCCLM3, HepG2, and Bel-7402 cells**. (A-C) Cell numbers at G_2_/M and S phases decreased dramatically in HCCLM3, HepG2, and Bel-7402 cells in a dose-dependent manner. (D-F) After exposure to 4, 16, and 64 mg/mL IMOS for 24 hours, the number of apoptotic cells of HCCLM3, HepG2, and Bel-7402 were significantly increased as compared with control cells. (G-I) After treatment for 48 hours, IMOS significantly induced apoptotic death in HCCLM3, HepG2, and Bel-7402 cells at doses from 16 to 64 mg/mL.

To address whether proliferation inhibition of IMOS was attributed to cell cycle arrest, cell cycle phases of HCCLM3, HepG2, and Bel-7402 cells were next analyzed by flow cytometry. As expected, cell numbers at the S and G_2_/M phases were significantly decreased, whereas cell numbers at the G_0_/G_1 _phase were markedly increased after IMOS treatment in a dose-dependent manner (Figure 3A-C). To further investigate whether cell apoptosis was also involved in proliferation inhibition caused by IMOS, early and late apoptotic cells were monitored by annexin V and PI staining, respectively. The percent of early apoptosis cells increased significantly after ≥4 mg/mL IMOS treatment (Figure 3D-F). The percent of late apoptotic cells was statistically higher in HCCLM3, HepG2, and Bel-7402 cells after ≥16 mg/mL IMOS treatment (Figure 3G-I).

### Adhesion, migration, and invasiveness of HCCLM3 inhibited by IMOS in vitro

**Figure 4:**
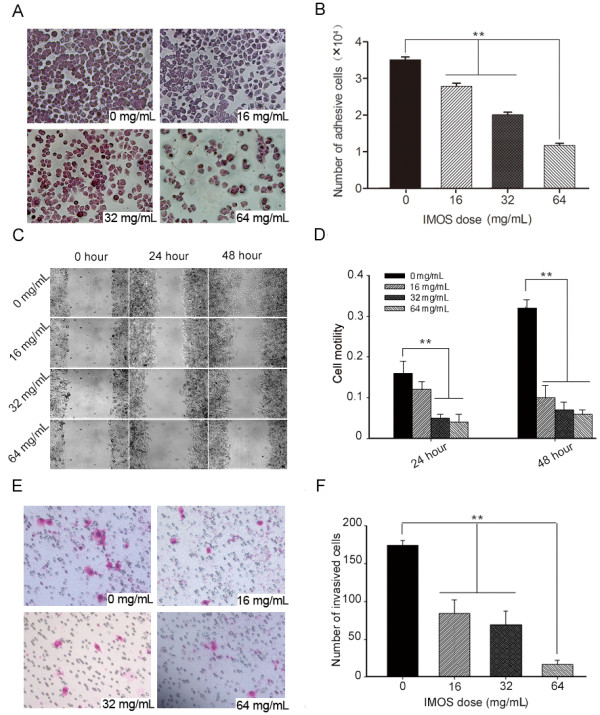
**Inhibitory effects of IMOS on HCCLM3 cell adhesion, migration, and invasiveness**. (A) Representative images of adhesion cell (×200) and (B) HCCLM3 adhesion was markedly inhibited by IMOS at doses of 16, 32, and 64 mg/mL as compared with sham treatment. (C) Representative images of migration distances (×100) and (D) HCCLM3 migrations began to be significantly inhibited by 16 mg/mL IMOS after a 24-hour treatment, and by 4 mg/mL IMOS after a 48-hour treatment (*P < 0.01*). (E) Representative images of transmembrane cells (×200) after treatment with 0, 16, 32, and 64 mg/mL IMOS. (F) Invasiveness of HCCLM3 was inhibited by IMOS in a dose-dependent manner.

To detect antitumor activities of IMOS on HCCLM3, cell adhesion, wound healing, and transwell assays were performed after treatment with IMOS at doses of 0, 16, 32, and 64 mg/mL, respectively. HCCLM3 adhesion was markedly inhibited by IMOS at doses of 16, 32, and 64 mg/mL as compared with sham treatment (Figure 4A and 4B). HCCLM3 migration was significantly suppressed in a dose- and time-dependent manner (Figure 4C and 4D). In addition, the numbers of transmembrane cells after IMOS treatment with doses of 0, 16, 32, and 64 mg/mL were 174.67 ± 5.69, 84.33 ± 18.15, 69 ± 18.52, and 17 ± 5.57, respectively (Figure 4E and 4F). These data demonstrate that IMOS is a potent inhibitor of cell adhesion, migration, and invasiveness of HCCLM3.

### Maximum tolerable dose of IMOS in vivo

To determine the maximum tolerable dose for in vivo therapeutic study, IMOS with an initial dose of 30 mg/kg/d was injected intraperitoneally into mice once daily in an escalating-dose schedule. No mouse death was observed during a 30-day treatment with IMOS given at 30, 60, and 90 mg/kg/d. On day 30, the numbers of thrombocytes in mice treated with IMOS at 0, 30, 60, and 90 mg/kg/day were 1164 ± 183, 1089 ± 210, 1170 ± 224, and 1049 ± 258 × 10^9^/L, respectively. No obvious thrombocytopenia was found after IMOS treatment. No significant abnormalities were found in these mice as evaluated by body weights (Additional file 2), hepatorenal function (Additional file 3), and pathologic examinations of heart, liver, and kidney tissues (Additional file 4). However, mice began to die at day 6 at an IMOS dose of 180 mg/kg/d. Therefore, IMOS at a dose of ≤90 mg/kg/d was well tolerated by athymic BALB/c mice.

### HCC growth and metastasis suppressed by IMOS in xenograft models

**Figure 5:**
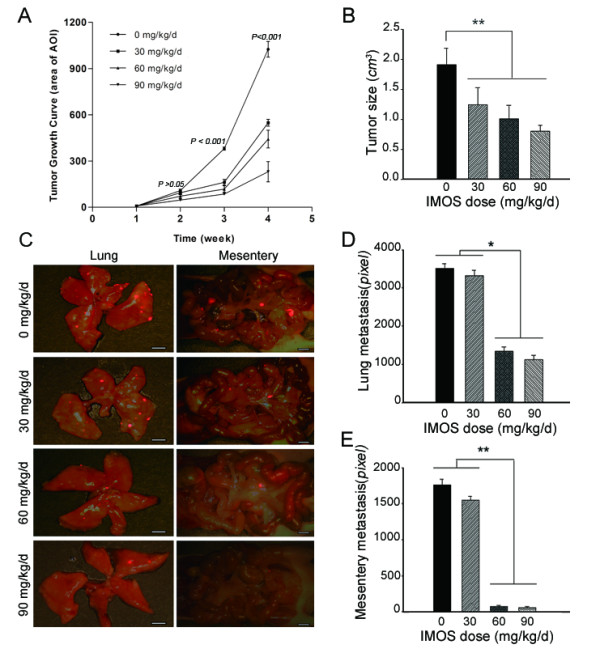
**Effects of IMOS on tumor growth and metastasis of HCCLM3-RFP xenograft**. (A) HCC growth was dramatically suppressed by IMOS treatment, especially at doses of 60 and 90 mg/kg/d after 3 weeks. (B) Tumor volumes in mice treated with IMOS at 30, 60, and 90 mg/kg/d were markedly smaller than the volume in sham-treated mice on day 30 (*P < 0.01*). (C-E) Lung and mesentery metastases were significantly inhibited by IMOS at doses of 60 and 90 mg/kg/d (*P < 0.01 *for both). (C) Representative fluorescent images and (D and E) statistical results of metastatic foci in lung and mesentery (*P < 0.01*).

To determine its effects on HCC progression, IMOS was intraperitoneally given to HCCLM3 xenograft mice at doses of 30, 60 and 90 mg/kg/d. In accord with the results in vitro, IMOS inhibited tumor growth and metastasis of HCCLM3 xenograft in a dose-dependent pattern (Figure 5A-E). Tumor growth was observed to be dramatically suppressed by IMOS at 90 mg/kg/d after a 3-week treatment (Figure 5A). On day 30, tumor volumes in 30, 60 and 90 mg/kg/d IMOS-treated mice were 1.24 ± 0.28 cm^3^, 1.01 ± 0.22 cm^3^, and 0.8 ± 0.1 cm^3^, respectively, much smaller than the volume in sham-treated mice (1.91 ± 0.27 cm^3^, *P *< 0.001; Figure 5B). Furthermore, metastatic foci in lung and mesentery in mice treated with IMOS doses of 30, 60, and 90 mg/kg/d were 3327 ± 137 and 1547 ± 56,1335 ± 115 and 72 ± 15, 1120 ± 105 and 60 ± 11, respectively, which were also statistically smaller than seen in sham-treated mice (3506 ± 125 and 1764 ± 78; Figure 5C-E). The results suggest that IMOS has potent suppressive activities not only on HCC growth but also on HCC metastasis.

### Signal pathways and gene expressions regulated by IMOS

**Figure 6:**
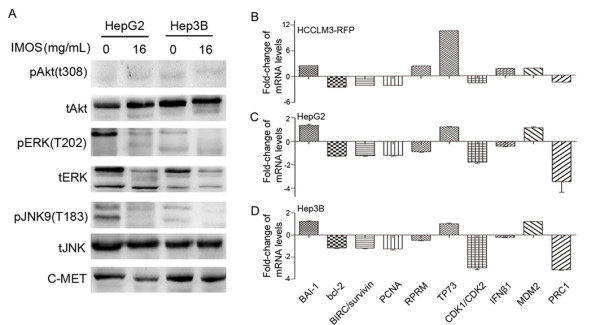
**Signaling pathways and gene expressions regulated by IMOS**. (A) pERK, tERK, and pJNK were significantly down-regulated by IMOS in both HepG2 and Hep3B cells, whereas c-MET was markedly down-regulated only in HepG2 cells. No obvious changes were found in protein levels of pAkt, tAkt, and tJNK. (B) Ten genes were significantly regulated by IMOS in HCCLM3-RFP xenografts. Among them, *Bcl-2*, *BIRC5/survivin*, *PCNA*, *CDK1/CDK2*, and *PRC1 *were down-regulated, and *BAI-1*, *TP73*, and *MDM2 *up-regulated by more than twice. (C,D) Except for *RPRM/Reprimo *and *IFN-β*, similar expression profiles were confirmed in IMOS-treated HepG2 and Hep3B cells.

To understand underlying mechanisms, total and phosphorylated protein levels of AKT, ERK, and JNK as well as c-MET were analyzed in sham-treated and 16-mg/mL IMOS-treated HepG2 and Hep3B cells. The levels of pERK, tERK, and pJNK were significantly down-regulated in both cell lines, whereas the level of c-MET was markedly down-regulated only in HepG2 cells. No obvious changes were found in the protein levels of pAkt, tAkt, and tJNK (Figure 6A). Furthermore, 10 differentially expressed genes were found in 90-mg/kg/d IMOS-treated xenograft tissues as compared with sham-treated tissues. Among them, *Bcl-2*, *BIRC/survivin*, *PCNA*, *CDK1/CDK2*, and *PRC1 *were down-regulated more than 2-fold, whereas *BAI-1*, *TP73*, and *MDM2 *were up-regulated more than 2-fold (Figure 6B). Except for *RPRM/Reprimo *and interferon β (*IFN-β*), similar expression profiles were confirmed in IMOS-treated HepG2 and Hep3B cells as compared with HCCLM3 xenograft (Figure 6C and 6D).

## Discussion

HCC usually has a limited response to current pharmacotherapy. It has been reported that MHCC97L and HepG2 cells surviving oxaliplatin treatment show enhanced migration and invasion in vitro and increase metastasis to the lung when reinoculated into nude mice [19]. Similar results have been observed in our previous study that IFN-α had contrasting aspects of consistently suppressing HCC growth but also promoting tumor metastasis capacity [20]. Therefore, it is urgent to investigate new agents with robust inhibitory effects on both HCC growth and metastasis. Fortunately, small molecular agents of sulfated oligosaccharides were confirmed to have potent antitumor activities against primary tumor growth and metastasis [11,12]. Therefore, IMOS was assumed to have similar activities against HCC progression. As expected, IMOS dramatically inhibited cell proliferation and induced cell cycle arrest and apoptosis in three tested HCC cell lines. Furthermore, suppressive effects on cell adhesion, motility, and invasiveness of HCCLM3 in vitro as well as tumor growth and metastasis of HCCLM3 xenograft in vivo were obviously achieved by IMOS treatment in a dose-dependent manner. These findings suggest that IMOS is a possible novel compound to be used against progression of HCC.

According to previous studies, sulfated oligosaccharides were thought to suppress tumor angiogenesis, growth, and metastasis primarily by their competitive inhibition of the cleavage of heparan sulfate-growth factor complex, thus reducing the release of growth factors, such as vascular endothelial growth factor and basic fibroblast growth factor from the microenvironmental matrix [21]. However, many direct actions of IMOS on HCC cells in vitro cannot be satisfactorily explained by its inhibition of heparanase activities. Therefore, we focused our mechanism studies on cell proliferation and apoptosis regulation in this study.

Numerous studies have shown that Akt, ERK, and JNK signaling pathways have important roles in cancerous cell proliferation, cell cycle, and apoptosis regulation in a *p53*-dependent or -independent manner [22-24]. Dysfunctions of those pathways are common events in tumorigenesis and progression in many types of cancers, including HCC [25,26]. Therefore, HepG2, a HCC cell line with wild-type *p53*, and Hep3B, a cell line with mutant *p53*, were used for further study to elucidate the mechanism of IMOS. Our preliminary results from Western blots revealed that ERK and JNK but not Akt signaling pathways were significantly inhibited by IMOS in both cell lines, whereas c-MET, a well-known *p53 *transcriptional target, was only down-regulated in HepG2 [27]. All those results indicated that ERK/JNK signaling pathways were involved in IMSO-mediated *p53 *activity in HepG2, but not in Hep3B.

Ten differentially expressed genes were screened from HCCLM3-RFP xenografts. Eight of them were confirmed in IMOS-treated HepG2 and Hep3B cells. Because similar profiles of most differentially expressed genes were found in both HepG2 and Hep3B cells, IMOS may inhibit cell proliferation and induce cell apoptosis of HCC in a *p53*-independent manner. Among them, *BAI-1 *and *TP73 *were significantly up-regulated, whereas *Bcl-2*, *BIRC5/survivin*, *PCNA*, and *CDK1/CDK2 *were markedly down-regulated. Our findings are consistent with previous observations that *Bcl-2*, *BIRC5/survivin*, and *BAI-1 *were the mediators of cell cycle arrest and apoptosis regulation [28-30]. Ectopic expression of *PCNA *was able to suppress cell apoptosis [31], thus decreased expression of *PCNA *in HCC after IMOS treatment was probably able to induce cell apoptosis. Furthermore, as an apoptosis-induced gene [32], enhanced expression of *P73 *in IMOS-treated tissue may also promote HCC apoptosis.

## Conclusions

IMOS, a novel sulfated oligosaccharide, at doses of ≤90 mg/kg/d exhibited potent antitumor effects on experimental HCC growth and metastasis. It should be a promising anti-HCC agent and worth further studies in patients with HCC, especially disease at advanced stages.

## Competing interests

The authors declare that they have no competing interests.

## Authors' contributions

CLX, ZHT, and JLW organized the study, analyzed the effects of IMOS on cell proliferation, apoptosis, adhesion, migration, and invasiveness, and helped to write the manuscript. LG contributed to the analysis of the hepatorenal functions and the number of platelets after IMOS treatment. WWL and JLW performed the statistical and cell signal pathway analysis. HCS and LW participated in the design and coordination of the study. ZYT, JF, and WZW contributed to the interpretation of the results and helped write the manuscript. All authors read and approved the final manuscript.

## Pre-publication history

The pre-publication history for this paper can be accessed here:

http://www.biomedcentral.com/1471-2407/11/150/prepub

## Supplementary Material

Additional file 1 **Primer sequences and amplification conditions of qRT-PCR**. Click here for file

Additional file 2**Body weights of nude mice after IMOS treatment (g,  ± SD)**.Click here for file

Additional file 3**Hepatorenal parameters of nude mice after IMOS treatment ( ± SD)**.Click here for file

Additional file 4 **Pathologic examinations of heart, liver, and kidney tissues after treatment with IMOS**. Click here for file
