# Supplementary material for: Isomalto oligosaccharide sulfate inhibits tumor growth and metastasis of hepatocellular carcinoma in nude mice
Source: BMC Cancer. 2011 Apr 22;11:150. doi: 10.1186/1471-2407-11-150 (PMC3107808; doi:10.1186/1471-2407-11-150)
Supplement: Additional file 1 — Primer sequences and amplification conditions of qRT-PCR. [file 1471-2407-11-150-S1.DOC]

| Gene ID | Gene name | Forward primer | Reverse primer | Annealing temperature |
| --- | --- | --- | --- | --- |
| NM_001702 | *BAI-1* | CTACATCCGCTGTGTTTCCAT | TCTCTGTCATGTTCCTCAGGAC | 60 |
| NM_000633 | *BCL-2* | GAACTGGGGGAGGATTGTGG | CCGGTTCAGGTACTCAGTCA | 59 |
| NM_001168 | *BIRC5/survivin* | AGGACCACCGCATCTCTACAT | AAGTCTGGCTCGTTCTCAGTG | 58 |
| NM_182649 | *PCNA* | ACACTAAGGGCCGAAGATAACG | CGGCATATACGTGCAAATTCAC | 58 |
| NM_019845 | *RPRM* | CTAGGCAACCAGACGGACGTG | CCCTCGGACTTGATGAGCAGA | 57 |
| NM_005427 | *TP73* | GGAACCAGACAGCACCTACTT | CTCAGCAGATTGAACTGGGC | 56 |
| NM_001786 | *CDK1/CDC2* | AGGTCAAGTGGTAGCCATGAA | ACAAAACACAATCCCCTGTAGG | 58 |
| NM_002176 | *IFNβ1* | CCAACAAGTGTCTCCTCCAAA | TCTCCTCAGGGATGTCAAAGT | 56 |
| NM_002392 | *MDM2* | AGCCTGGCTCTGTGTGTAATA | CCTGATCCAACCAATCACCTGA | 57 |
| NM_003981 | *PRC1* | TCGATTTACAAACCGAGGAGGA | CCCACAATTCAATTCGTGCCTT | 58 |

Additional file 1: Primer sequences and amplification conditions of qRT-PCR
