# Supplementary material for: Isomalto oligosaccharide sulfate inhibits tumor growth and metastasis of hepatocellular carcinoma in nude mice
Source: BMC Cancer. 2011 Apr 22;11:150. doi: 10.1186/1471-2407-11-150 (PMC3107808; doi:10.1186/1471-2407-11-150)
Supplement: Additional file 2 — Body weights of nude mice after IMOS treatment (g, ± SD). [file 1471-2407-11-150-S2.DOC]

Additional file 2: Body weights of nude mice after IMOS treatment（g, ±SD）

| *Time*  *(Week)* | Dosage (mg/kg/d) | | | | *P* Value***** |
| --- | --- | --- | --- | --- | --- |
|  | 0 | 30 | 60 | 90 |  |
| 0 | 18.42±1.11 | 18.72±1.02 | 18.70±1.02 | 19.38±0.45 | 0.150 |
| 1 | 19.67±1.16 | 20.30±0.85 | 20.68±0.90 | 20.83±1.19 | 0.105 |
| 2 | 23.20±1.15 | 23.50±0.87 | 23.80±0.84 | 23.79±0.79 | 0.620 |
| 3 | 24.20±1.33 | 25.00±1.01 | 25.30±1.41 | 25.50±0.88 | 0.155 |
| 4 | 25.20±1.54 | 26.10±1.34 | 26.40±1.45 | 26.70±1.64 | 0.254 |

NOTE. * One-way ANOVA. n=10.
