# Supplementary material for: Isomalto oligosaccharide sulfate inhibits tumor growth and metastasis of hepatocellular carcinoma in nude mice
Source: BMC Cancer. 2011 Apr 22;11:150. doi: 10.1186/1471-2407-11-150 (PMC3107808; doi:10.1186/1471-2407-11-150)
Supplement: Additional file 3 — Hepatorenal parameters of nude mice after IMOS treatment ( ± SD). [file 1471-2407-11-150-S3.DOC]

Additional file 3: Hepatorenal parameters of nude mice after IMOS treatment（±SD）

| Parameters | Dosage (mg/kg/d) | | | | *P*  Value* |
| --- | --- | --- | --- | --- | --- |
|  | 0 | 30 | 60 | 90 |  |
| Glu（mmol/L) | 7.22±0.41 | 7.18±0.83 | 6.74±0.74 | 6.70±0.24 | 0.251 |
| Crea(μmol/L) | 17.30±1.81 | 18.70±3.13 | 19.40±3.60 | 17.00±2.00 | 0.311 |
| Bun(mmol/L) | 8.06±1.19 | 7.98±1.11 | 7.84±0.56 | 7.16±0.96 | 0.431 |
| Ua（μmol/L) | 270.00±69.20 | 229.00±64.10 | 303.00±39.30 | 285.00±45.00 | 0.085 |
| Tbil(μmol/L) | -0.35±0.64 | 0.44±0.33 | -0.70±0.89 | -0.20±0.00 | 0.087 |
| Alt(U/L) | 50.25±2.87 | 52.40±13.30 | 44.25±15.92 | 45.00±7.07 | 0.730 |
| Ast(U/L) | 130.00±18.71 | 131.20±18.09 | 176.00±39.33 | 135.00±18.38 | 0.081 |
| Alp(U/L) | 130.50±11.47 | 117.20±5.22 | 119.25±28.32 | 119.00±7.07 | 0.655 |
| Ggt(U/L) | 2.89±0.78 | 2.33±1.22 | 3.43±1.13 | 2.00±1.22 | 0.115 |
| LDH(U/L) | 1130.50±314.02 | 1112.00±261.47 | 1493.25±242.66 | 1524.00±212.13 | 0.327 |
| Dbil(μmol/L) | -0.06±0.27 | 0.04±0.16 | -0.31±0.47 | -0.08±0.23 | 0.131 |

NOTE. * One-way ANOVA. n=10.
