# Supplementary figures and images for: Isomalto oligosaccharide sulfate inhibits tumor growth and metastasis of hepatocellular carcinoma in nude mice
Source: BMC Cancer. 2011 Apr 22;11:150. doi: 10.1186/1471-2407-11-150 (PMC3107808; doi:10.1186/1471-2407-11-150)

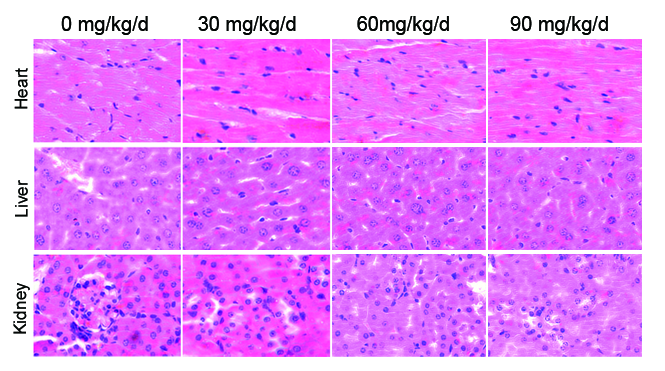

Supplement: Additional file 4 — Pathologic examinations of heart, liver, and kidney tissues after treatment with IMOS. [file 1471-2407-11-150-S4.TIFF]
